# Increasing Adherence to Prophylactic Swallowing Exercises During Head and Neck Radiotherapy: The Multicenter, Randomized Controlled PRESTO-Trial

**DOI:** 10.1007/s00455-022-10513-6

**Published:** 2022-09-19

**Authors:** Margot Baudelet, Fréderic Duprez, Leen Van den Steen, Sandra Nuyts, Daan Nevens, Ann Goeleven, Caroline Vandenbruaene, Hanne Massonet, Alice Vergauwen, Heleen Bollen, Sarah Deschuymer, Kristien Wouters, Marc Peeters, Carl Van Laer, Steven Mariën, Michiel Van den Brekel, Lisette van der Molen, Tom Vauterin, Joost van Dinther, Hilde Verstraete, Isabel Hutsebaut, Sabine Meersschout, Olivier Vanderveken, Marc De Bodt, Gwen Van Nuffelen, members of the Belgian PRESTO-group

**Affiliations:** 1 Faculty of Medicine and Health Sciences, University of Ghent, Ghent, Belgium; 2 Faculty of Medicine and Health Sciences, University of Antwerp, Universiteitsplein 1 Wilrijk, 2610 Antwerp, Belgium; 3 Department of Radiation Oncology, Ghent University Hospital, Corneel Heymanslaan 10, 9000 Ghent, Belgium; 4 Antwerp University Hospital, Antwerp, Belgium; 5 University Hospital Leuven, Louvain, Belgium; 6 KU Leuven, Louvain, Belgium; 7 Multi-Disciplinary Oncology Center Antwerp, Antwerp, Belgium; 8 Iridium Network, Antwerp, Belgium; 9 AZ Sint-Jan Brugge, Brugges, Belgium; 10 Clinical Trial Center (CTC), CRC Antwerp, Antwerp University Hospital, University of Antwerp, Edegem, Belgium; 11 Department Medical Oncology, Antwerp University Hospital, Antwerp, Belgium; 12 Department of Otolaryngology and Head and Neck Surgery-Rehabilitation Center for Communication Disorders, Antwerp University Hospital, Antwerp, Belgium; 13 Department of Head and Neck Oncology and Surgery, Antoni Van Leeuwenhoek, Netherlands Cancer Institute, Amsterdam, The Netherlands; 14 Department of ENT-HNS, European Institute for ORL-HNS, Sint-Augustinus Hospital, GZA, Antwerp, Belgium

**Keywords:** Dysphagia, Deglutition, Deglutition disorders, Head-and-neck cancer, Adherence, Prophylactic swallowing exercises, (Chemo)radiotherapy, Telehealth application

## Abstract

**Background:**

Prophylactic swallowing exercises (PSE) during radiotherapy can significantly reduce dysphagia after radiotherapy in head and neck cancer (HNC). However, its positive effects are hampered by low adherence rates during the burdensome therapy period. Hence, the main goal of this multicenter randomized controlled trial (RCT) was to investigate the effect of 3 different service-delivery modes on actual patients’ adherence.

**Methods:**

A total of 148 oropharyngeal cancer patients treated with primary (chemo)radiotherapy were randomly assigned to a 4 weeks PSE program, either diary-supported (paper group; *n* = 49), app-supported (app group; *n* = 49) or therapist-supported (therapist group; *n* = 50). Participants practiced 5 days/week, daily alternating tongue strengthening exercises with chin tuck against resistance exercises. Adherence was measured as the percentage of completed exercise repetitions per week (%reps). Statistical analysis was performed by means of SPSSv27, using Linear Mixed-effects Models with post hoc pairwise testing and Bonferroni-Holm correction.

**Results:**

Adherence and evolution of adherence over time was significantly different between the three groups (*p* < .001). Adherence rates decreased in all three groups during the 4 training weeks (*p* < .001). During all 4 weeks, the therapist group achieved the highest adherence rates, whilst the app group showed the lowest adherence rates.

**Conclusions:**

PSE adherence decreased during the first 4 radiotherapy weeks regardless of group, but with a significant difference between groups. The therapist group achieved the highest adherence rates with a rather limited decline, therefore, increasing the face-to-face contact with a speech-language therapist can overcome the well-known problem of low adherence to PSE in this population.

**Trial Registration:**

Trial registration: ISRCTN, ISRCTN98243550. Registered December 21, 2018 – retrospectively registered, https://www.isrctn.com/ISRCTN98243550?q=gwen%20van%20nuffelen&filters=&sort=&offset=1&totalResults=2&page=1&pageSize=10&searchType=basic-search.

## Background

Prophylactic swallowing exercises (PSE) in patients with head and neck cancer (HNC) undergoing radiotherapy or concomitant chemoradiotherapy (RT/CRT) have a significant positive effect on muscle condition, swallowing function and quality of life (QoL) [1–6]. Since dysphagia can lead to malnutrition, aspiration and related co-morbidities in HNC patients, prevention of this common side-effect is essential to increase QoL and long-term survival and thus to limit the load on our healthcare resources [1–3, 7–9]. Especially, since the number of surviving patients is increasing, due to improvements in diagnostics, treatment modalities such as concomitant CRT, and the increase of human papillomavirus (HPV)-related oropharyngeal cancer (OPC) patients, prevention of late sequelae becomes paramount [10].

Although PSE is known to significantly reduce dysphagia in HNC patients treated with RT/CRT, adherence rates are generally low (13%) to moderate (71%) [9, 11, 12]. This compromises the favorable effects the exercises have. Previous research has already shown that being adherent to PSE during RT/CRT is essential to benefit from the positive effects on swallowing function [13, 14]. In general, adherence can be improved by the use of continuous supervision, feedback after successful exercise performance and a close relationship between the patient and therapist [15, 16]. This indicates that the way the exercises are delivered, the service-delivery mode, can influence the degree of adherence [15, 17, 18]. Although little studied in HNC patients, this service-delivery mode might also impact on adherence to PSE in this population [19, 20].

Most commonly reported service-delivery modes for PSE are diary-supported home practice, app-supported home practice and speech and language pathologist (SLP)-supported practice [1, 2, 8, 19, 21]. The first option, diary-supported PSE, involves little additional cost and gives the patients the opportunity to practice whenever they want. Keeping a diary allows the therapist to monitor the exercises, thereby helping to increase adherence [20]. In app-supported PSE, the second option, telehealth applications are used to guide patients through home practice, without SLP-supervision of the therapy sessions. These are considered to be possible tools to improve traditional health care with better exercise adherence rates [22, 23]. Previous research showed that telepractice models to deliver speech-language therapy interventions are feasible in HNC patients and that they would be helpful to implement intensive rehabilitation in routine practice [8, 19, 23]. The third option, therapist-supported PSE, has the advantage of having continuous supervision and motivational support by an SLP, which has already been shown to improve adherence [20, 24].

Since the need for the development of an effective adherence improving PSE-program is crucial and internationally recognized [8, 11], the aim of this multicenter randomized controlled trial (RCT) was to investigate the effect of specific adherence improving measures on patients’ actual adherence to PSE. Adherence was examined across the three before mentioned service-delivery modes: (1) diary-supported PSE, (2) app-supported PSE and (3) therapist-supported PSE.

## Materials and Methods

### Study Design and Participants

The PRophylactic Swallowing Exercise Therapy program for patients with Oropharyngeal cancer (PRESTO) was a multicenter, open-label randomized controlled trial. Patients were enrolled at four Belgian hospitals (University Hospitals of Antwerp, Ghent and Leuven and General Hospital Sint-Jan Bruges).

Patients with a stage III or IVA-B (TNM7) newly diagnosed squamous cell carcinoma of the oropharynx were prospectively recruited for this study. Candidates for enrollment were both men and woman older than 18 years, with no cognitive or language deficits that might interfere with the correct implementation of the swallowing therapy protocol. Patients were treated with 6–7 weeks fractionated RT/CRT with or without induction chemotherapy. Exclusion criteria were the presence of a recurrent carcinoma or metastasis from a non-HNC carcinoma and previous RT/CRT or surgery in the head-neck region with possible impact on swallowing function. Written informed consent was obtained from all participants.

**Fig. 1:**
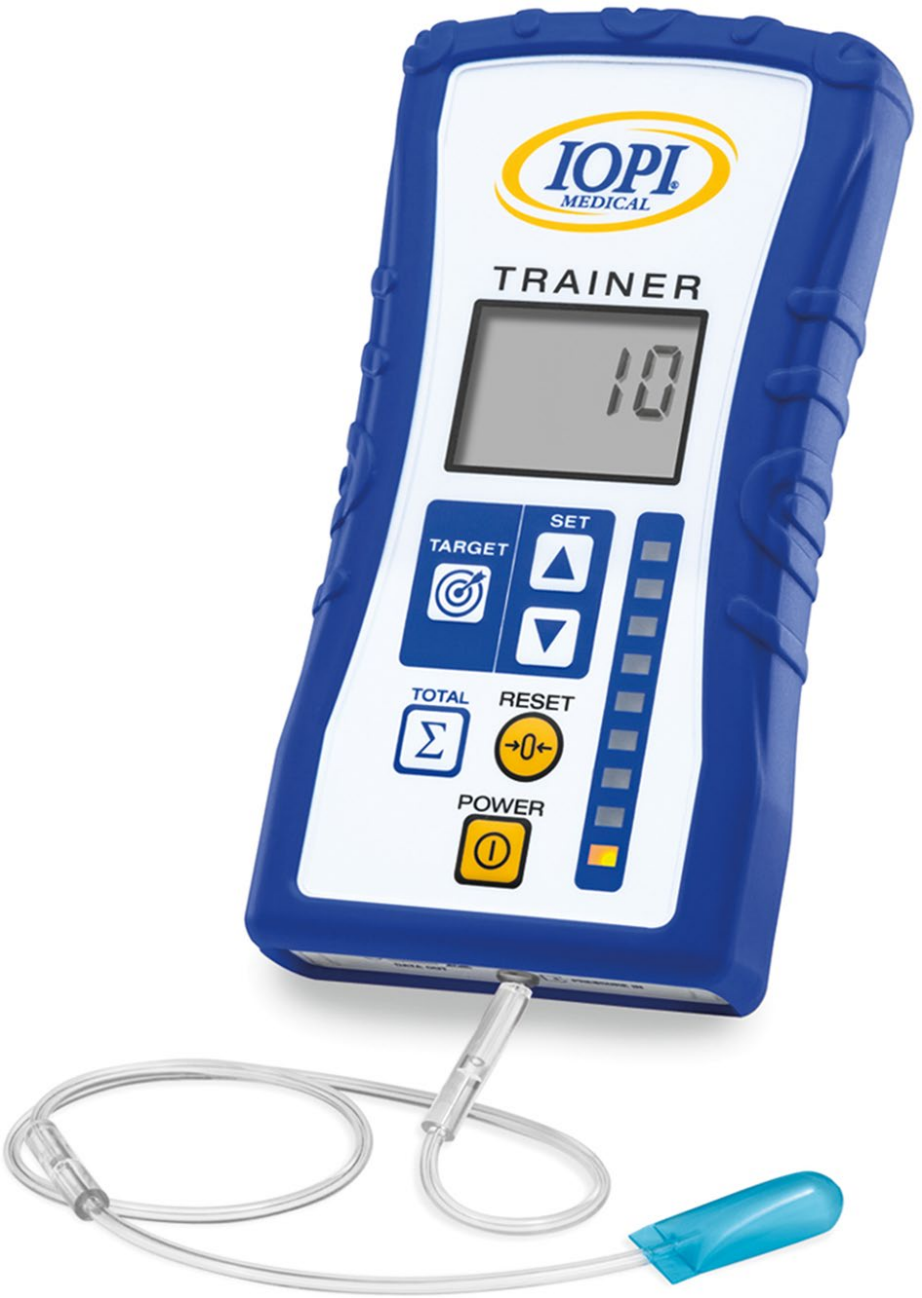
Iowa Oral Performance Instrument (model 3.2, IOPI Medical LLC, Woodinville, WA, USA)

**Fig. 2:**
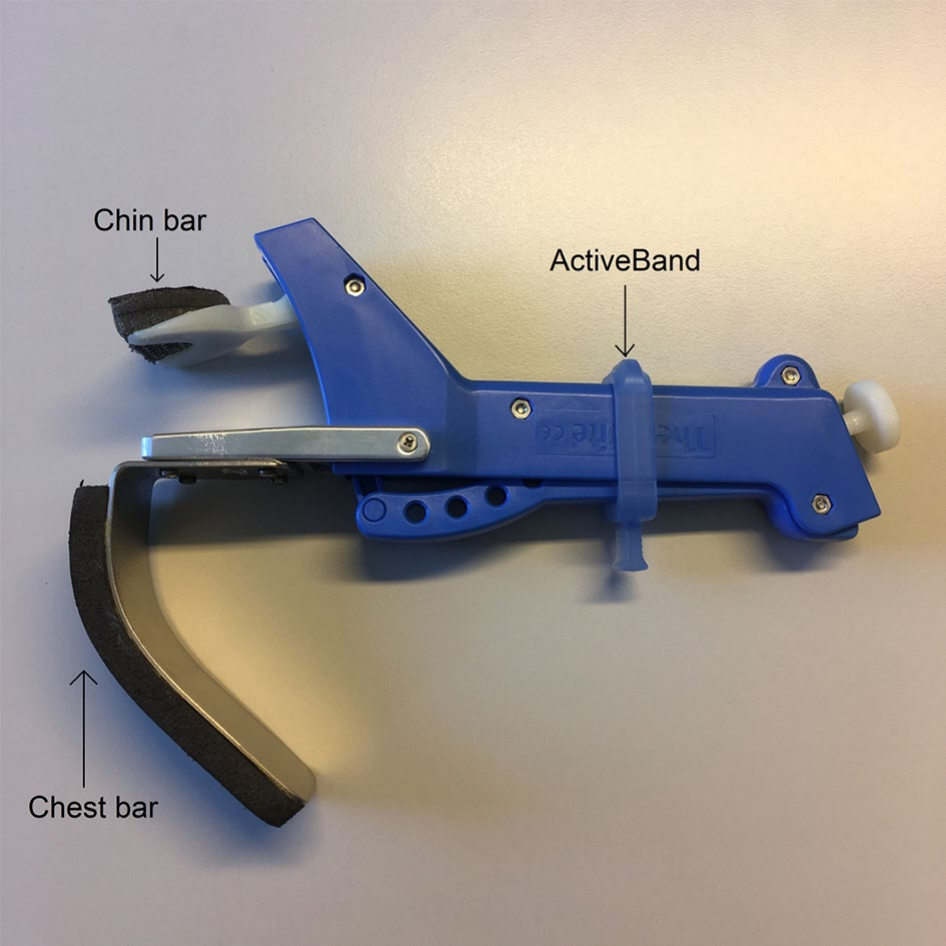
Swallowing Exercise Aid (Antoni Van Leeuwenhoek, Netherlands Cancer Institute)

The minimization program QMinim was used to randomly assign all participants with a 1:1:1 allocation to one of the three exercise groups: diary-supported PSE (paper group), app-supported PSE (app group) and therapist-supported PSE (therapist group). The minimization factors were age (20–60 vs. ≥ 60 years old), treating center, presence of baseline dysphagia and treatment (radiotherapy or concomitant chemoradiotherapy). The three groups differed in degree and kind of adherence-improving measures. All patients performed the same PSE program 5 times a week during the first 4 weeks of RT/CRT. Limiting the therapy program to the first 4 weeks of RT/CRT was a general measure to maximize adherence since acute toxicity typically peaks during week 5 of radiotherapy treatment [25, 26]. The PSE comprised 2 evidence-based exercises, alternating daily and targeting the main muscle groups involved in swallowing. First, tongue strengthening exercises (TSE) were done since tongue strength is the main bolus-driving force. Furthermore, reduced tongue strength can cause oral and pharyngeal residue and aspiration [27]. The TSE were performed using the Iowa Oral Performance Instrument (IOPI, model 3.2, IOPI Medical LLC, Woodinville, WA, USA) (Fig. 1) and consisted of 120 tongue presses per session, divided into 12 sets of 10 repetitions. Patients were instructed to pause for 30 s between every set. Second, chin-tuck against resistance (CTAR) exercises were used since they have a significant impact on the suprahyoid muscles [28]. The exercises were done using the Swallowing Exercise Aid (SEA) [28] (Fig. 2) and one session consisted of 30 sets of 5 repetitions for a total of 150 chin-tucks per day. Fifteen seconds of rest was provided between sets. The fifth repetition was a combination of a chin-tuck with an effortful swallow since this exercise has been shown to improve tongue-base posterior motion and tongue-base pharyngeal wall pressures [29]. Patients practiced at 60–80% of their 1repetition maximum (1RM), which was recalculated every week [30]. We practiced following the overload principle and took into account a high intensity [31].

### Adherence

The degree of adherence was expressed as the percentage of completed repetitions per week (%reps) and by means of 4 different categories, defined by Wall et al., including negligible practice (< 25%reps), low practice (25–50%reps), moderate practice (50–75%) and high practice (> 75%) [19]. At the end of the session, all patients, regardless of their group, were questioned about the degree of difficulty they had in completing the task, about the factors contributing to this difficulty and if they had any concerns or suggestions.

### Paper Group

The paper group performed the first exercise session under supervision of the SLP. After this first session, the IOPI and SEA were given home, patients received a logbook with written instructions and they continued practicing at home. They were asked to register in their logbook how many exercise repetitions they performed each day and whether these repetitions were successful or not. Adherence was then calculated based on the patients’ record.

Once a week, an appointment with the SLP was scheduled to recalculate the target level, based on their new 1RM.

### App Group

Similar to the paper group, the app group performed the first exercise session together with the SLP. During this first session, participants were coached in the use of the tablet and app. Both the tablet and exercise devices were then given home so that further practice could be done at home. The app allowed registering all repetitions and whether they were successful or not. Each week, an appointment with the SLP was scheduled to recalculate the target level and to read data from the tablet. Adherence was calculated based on this data.

The application was developed in collaboration with the 3D animation, app and game studio Cyborn, Antwerp, Belgium (https://www.cyborn.be). It was provided to patients on a SAMSUNG GALAXY TAB A6 and practicing at home didn’t require internet connection. Every week, the SLP connected the tablet with Wi-Fi to enable synchronization and data upload to the server.

**Fig. 3:**
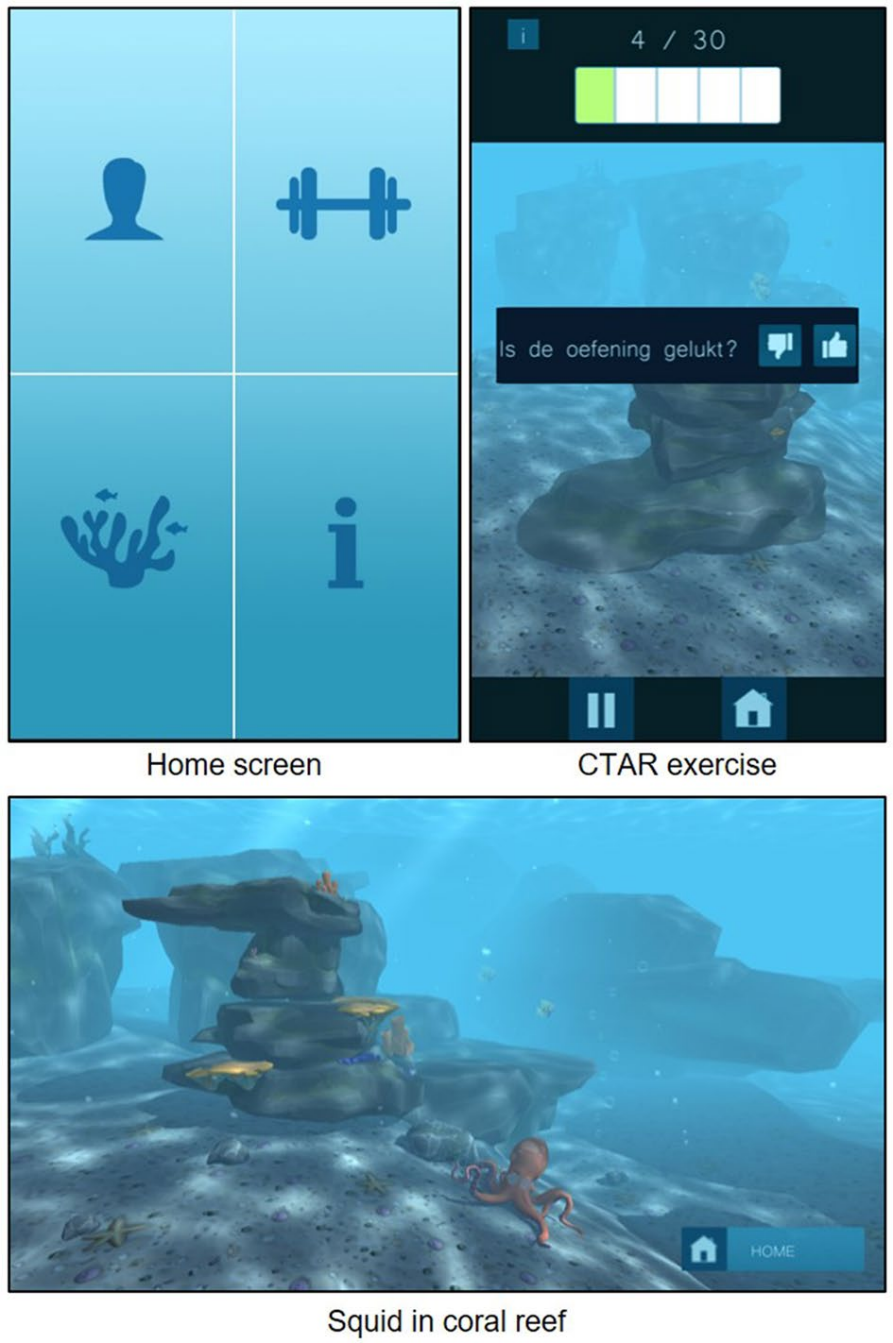
The application

The app consisted of instructional videos and images for both exercises. Patients could watch them as many times as needed to fully understand the exercise. The app uses gamification, i.e. using game elements in a non-game environment, with the aim of making a difficult task easier and more pleasant [32]. By means of gamification, it was expected that the app would help, support and motivate patients during practice. The aim of the game was to help a squid to make her underwater world more beautiful. Every time the patient practiced, he/she could win plants, flowers, stones and fish to brighten up the squids’ coral reef. Figure 3 shows screenshots of the application. The number of exercises and repetitions the patients completed, were saved.

Every week, patients in the app group were questioned about their experiences with the tablet and application using visual analogue scales (VAS). These scales were added in a later phase of the study, meaning that only a subset of patients systematically completed these questions. The VAS were 100 mm lines on which the patients were asked to place a vertical mark to indicate to what extent they agreed with the statement. The distance to the vertical mark was then measured to create a score. Table 1 shows an overview of the all scales concerning the tablet and app.

**Table 1:** Overview of VAS concerning the tablet and app

*User-friendliness*	
Not user-friendly	Very user-friendly
*Starting and opening of the app*	
Difficult	Easy
*Clarity of the explanation*	
Unclear	Very clear
*Usability of the explanation*	
Unusable	Very useful
*Layout of the app*	
Unattractive	Attractive
*Added value of the game element*	
No added value	Definitely added value
*Adequacy of the game element*	
Not adequate	Very adequate

### Therapist Group

The patients in the therapist group were given face-to-face therapy for 5 days/week. Each session, clear and repeated instructions were given and patients received continuous feedback on their performance by the SLP. The therapist kept a logbook and registered the number of exercise repetitions the patients performed each day, and whether these repetitions were successful or not. Adherence was calculated based on therapist data.

The full study protocol has been published previously [33].

### Statistical Analysis

#### Sample Size Calculation

The sample size calculation was performed for the primary outcome, namely MASA-C, using GLIMMPSE online software for power calculation in linear mixed effects models. No power calculation was done for the outcome adherence. The targeted total sample size, taking into account 20% dropouts, was 150; i.e. 50 patients per group. More details on the sample size calculation are presented in the protocol publication [33].

#### Data Analysis

Descriptive statistics were used to summarize patient characteristics per treatment group. A linear mixed-effects model with group, time and group by time interaction as fixed effects was used to study the evolution of adherence in the three groups. A random intercept per subject was added to account for the correlation between observations coming from the same individual. Time was considered categorical to be able to capture a non-linear evolution over time. Post hoc pairwise testing with Bonferroni-Holm correction for multiple testing was performed. As the adherence rates were not normally distributed, with a peak at 0% and 100%, an additional sensitivity analysis using ordinal logistic generalized estimating equations (GEE) with the 4 categories defined by Wall et al. (high, moderate, low, negligible practice) was performed.

We hypothesized that there would be a significant difference in adherence between the three groups, with the highest levels of adherence found in the therapist group, followed by the app group and that the lowest adherence rates would be found in the paper group. Attitudes towards exercises were analyzed by means of descriptive statistics. A linear mixed-effects model was also used to examine the effect of age and patients’ experiences towards mobile phones and apps on adherence in the app group.

Data was assumed to be missing at random and this missingness was ignored in the analyses. In the linear mixed effects model all information on the available time points is incorporated. Since only 11.4% of data was missing, we did not perform a sensitivity analysis using multiple imputation, as described in our statistical analysis plan [33].

A *p*-value of less than 0.05 was considered statistically significant. All analyses were conducted using SPSS Statistics version 27 (IBM, Chicago, IL, USA). Statistical analysis was performed under the supervision of a biostatistician.

## Results

### Participants

One hundred and fifty patients were recruited for this study. In one patient, baseline measurements were never taken due to an acute life-threatening hospitalization. Another patient was excluded due to a change in the study protocol, namely by adding the exclusion criteria of having a tracheotomy influencing the execution of the CTAR exercise. This leaves us with a total cohort of 148 patients. Patients, disease and treatment characteristics of the whole cohort and separate groups can be found in Table 2. One patient had multiple primary tumors, here the larger T-stage was taken into account in the calculations.

**Table 2:** Patients, disease and treatment characteristics

	Total cohort *n* = 148 (%)	Paper group *n* = 49 (%)	App group *n* = 49 (%)	Therapist group *n* = 50 (%)
Age	M = 63 SD = 8.5 Range = 41-86	M = 63 SD = 9.5 Range = 41-86	M = 63 SD = 7.9 Range = 41-83	M = 63 SD = 8.2 Range = 45-80
Gender Female Male	35 (24) 113 (76)	14 (29) 35 (71)	11 (22) 38 (78)	10 (20) 40 (80)
T classification 1-2 3-4	75 (51) 73 (49)	25 (51) 24 (49)	22 (45) 27 (55)	28 (56) 22 (44)
N classification 0 1 2-3	7 (5) 23 (15) 118 (80)	3 (6) 7 (14) 39 (80)	3 (6) 7 (14) 39 (80)	1 (2) 9 (18) 40 (80)
Treatment RT CRT CRT with induction CT	21 (14) 102 (69) 25 (17)	6 (12) 37 (76) 6 (12)	8 (16) 32 (65) 9 (19)	7 (14) 33 (66) 10 (20)
HPV status Positive Negative	76 (51) 72 (49)	24 (49) 25 (51)	23 (47) 26 (53)	29 (58) 21 (42)

M mean, SD standard deviation, RT radiotherapy, CRT chemoradiotherapy, CT chemotherapy

Patients who immediately dropped out after baseline measures (paper group: *n* = 1, app group: *n* = 3, therapist group: *n* = 1) were not included in the adherence analyses, giving a final number of 48 patients in the paper group, 46 in the app group, and 49 in the therapist group. Table 3 shows an overview of all drop-outs with the timing of drop-out and reason for drop-out. During the exercise weeks, there were 9 drop-outs in the paper group, 8 drop-outs in the app group and 4 drop-outs in the therapist group. As shown in Table 3, a total of 18 patients refused further participation in the study. The most common reasons were pain, general weakness and the additional burden the exercises put during the RT/CRT. One patient refused further participation because of disbelief in the exercises.

**Table 3:** Overview of drop-outs

	Timing						Reason
	Before start*	During week 1	During week 2	During week 3	During week 4	After week 4	
Paper group (n = 11)	1	5	2	-	2	1	•Hospitalization with impossibility of continuing exercises (n = 2) •Refuse to further participation (n = 8) •Died (n = 1)
App group (n = 13)	3	3	3	2	-	2	•Acute hospitalization with impossibility of continuing exercises (n = 1) •Progressive disease (n = 1) •Refuse to further participation (n = 8) •Died (n = 3)
Therapist group (n = 6)	1	1	2	1	-	1	•Acute hospitalization with impossibility of continuing exercises (n = 1) •Refuse to further participation (n = 2) •COVID-19 infection for which applicable rules required stop of study contacts (n = 2) •Submental swelling (n = 1)

*Excluded from the statistical analysis

### Adherence

**Fig. 4:**
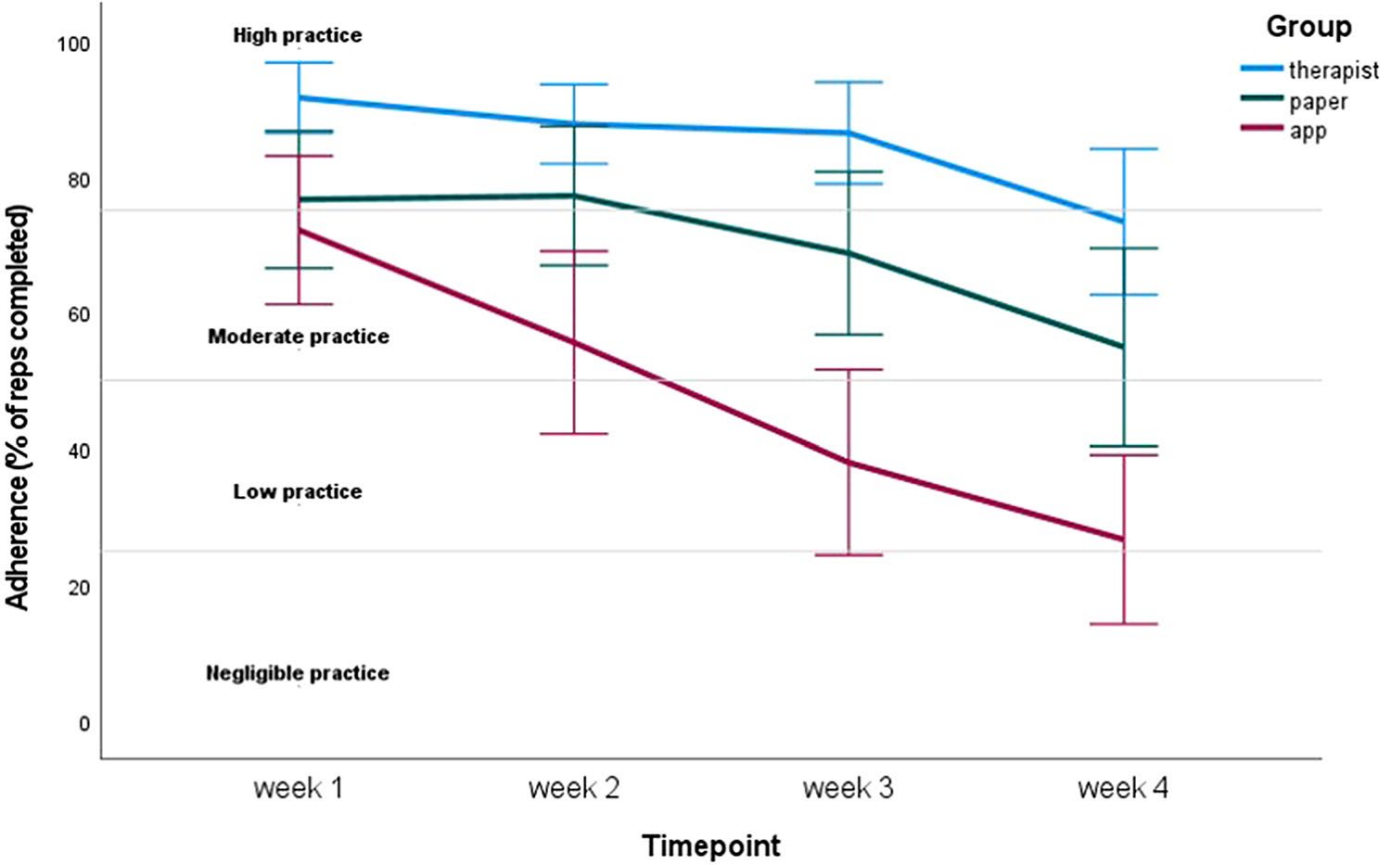
Adherence rates through time by service-delivery mode. Levels of adherence by Wall et al. (2016) applied

Linear mixed-effects models showed significant effects of group (F_(2, 119)_ = 20.194, *p* < 0.001), time (F_(3, 342)_ = 43.988, *p* < 0.001) and group by time interaction (F_(6, 342)_ = 4.546, *p* < 0.001). These effects were confirmed by the additional GEE analysis. Adherence rates decreased in all 3 groups during the 4 training weeks, but with significant differences between groups. The highest decline was found in the app group and the smallest decline in the therapist group. During all 4 weeks, the therapist group achieved the highest adherence rates (Fig. 4). Between week 1 and 4, adherence rates decreased significantly in the paper group from 77%reps to 55%reps; from 72%reps to 27%reps in the app group and from 92%reps to 73%reps in the therapist group. Post hoc analyses, adjusted by means of Bonferroni-Holm correction, were performed and are shown in Table 4. Adherence rates during week 1 and 2 were already significantly lower in the app group compared to the therapist group and during week 2 the paper group reached significantly higher adherence rates than the app group. During week 3 and 4, there was a significant difference between each of the three groups: adherence was significantly higher in the paper group compared to the app group and patients in the therapist group achieved significantly higher adherence rates than patients in the other 2 groups.

**Table 4:** Post hoc comparisons between therapy groups based on linear mixed effects model for %reps with Bonferroni Holm correction for multiple testing

	Paper group vs. app group			Paper group vs. therapist group			App group vs. therapist group		
	*p*	Difference (M%)	95% CI	*p*	Difference (M%)	95% CI	*p*	Difference (M%)	95% CI
W1	.539	4.51	[-9.93-18.93]	.099	14.95	[-28.68- -1.22]	.035	19.46	[-33.53- -5.37]
W2	.035	21.55	[5.46-35.69]	.112	10.56	[-28.20-.37]	<.001	32.11	[-49.11- -19.88]
W3	.002	30.68	[13.98-44.32]	.025	17.67	[-35.47- -6.70]	<.001	48.35	[-64.89- -35.58]
W4	.006	28.18	[11.27-41.99]	.025	18.40	[-36.00- -7.11]	<.001	43.58	[-62.96- -33.40]

W1, week 1; W2, week2; W3, week 3; W4, week 4; M%, mean percentage; CI, confidence interval

### Levels of Adherence

Wall and colleagues [19] defined 4 levels of adherence to PSE, including negligible practice (< 25%reps), low practice (25–50%reps), moderate practice (50–75%) and high practice (> 75%). In Fig. 4, we applied those levels of adherence on current results. The adherence in the therapist group can be labeled as high during the first 3 weeks and moderate during week 4 (73%). The paper group reached high adherence rates during week 1 and 2 and moderate rates during week 3 and 4, whereas the app group had moderate (week 1 and 2) to low (week 3 and 4) adherence rates.

### Influence of Age and Digital Experience in the App Group

When dividing the app group into two age groups, < 60 years old and > 60 years old, no significant differences were found in adherence rates (F_(1, 135)_ = 1.712, *p* < 0.193). Moreover, there was no impact of the degree of experience the patients had with tablets/mobile apps on patients’ adherence (F_(2, 135)_ = 0.049, *p* = 0.952).

In 13 of the 46 patients in the app group, the attitudes towards the tablet and app were systematically questioned. Table 5 gives an overview of the results of the visual analogue scales. Overall, the tablet and app were judged to be user-friendly, the app startup was found easy and the app was found to be very clear and useful. However, the added value of the game element and its adequacy concerning age, gender, etc. were scored less strong.

**Table 5:** Results of VAS concerning attitudes and experiences towards tablet/app (n = 13)

	Usability tablet	Opening + starting app	Transparency explanations and videos	Usability explanations and videos	Lay-out app	Added value game element	Adequacy of game
M (SD)	85 (13.7)	93 (10.0)	92 (11.8)	90 (13.2)	86 (15.1)	59 (36.4)	57 (35.5)

## Discussion

This multicenter RCT is only the second to investigate the effect of service-delivery mode on adherence to PSE in patients with oropharyngeal cancer. We found a significant impact of service-delivery mode (therapist, paper, app), time (1–4 weeks of RT) and the interaction of both on adherence.

During the 4 training weeks, adherence rates decreased significantly, which is consistent with previous studies [19, 20, 25, 34]. Messing and colleagues showed that adherence to PSE decreases rapidly during RT and drops to very low levels by the end of the treatment [25]. This remarkable decrease reflects the effect of RT-induced acute toxicity, which, as often described in literature, kicks in the second or third week of radiotherapy, peaks during the fifth week and lasts until the end or even after treatment [20, 25].

Although overall adherence rates in PRESTO decrease, they were found to be higher than the reported rates by Wall et al. [19]. In addition, the adherence rates in our paper-group were also higher than the rates in the study of Messing et al. [25]. Possible reasons for this discrepancies are differences in therapy content as well as organizational issues. Firstly, both Messing and Wall used a battery of exercises, while current study only consisted of two different types of exercises, which in addition, alternated daily [19, 25]. Previous research in home-based physical therapy showed that higher adherence rates can be achieved when limiting the prescribed exercises to only two different types [35]. This finding can also be applied to our results. Next to that, the treatment period in current study was limited to the first 4 weeks of RT/CRT, taking into account the well-known problem of peaking acute toxicities during week 5 [25, 26]. The idea was to maximally strengthen the patients’ swallowing system, before the patients suffer severely from the acute toxicities. We hypothesized that it might be mentally easier for the patients when they know the PSE will only last for the first four weeks and not until the end of the RT/CRT period, which might seem hardly reachable at the beginning. Note that due to differences in adherence definitions, caution is warranted when comparing different studies [12].

Current results show that adherence rates depend on service-delivery mode, with the highest adherence rates found in the therapist group. This highlights the positive effect of a combination of continuous supervision, feedback on performance and a close relationship between patient and therapist. These findings are both in line with results found within physical therapy research and with previous findings towards adherence rates to PSE in HNC patients [15, 17–20]; Hajdú et al. suggested that frequent supervision has a decisive effect on adherence (20). Compared to the therapist group, lower adherence rates were found in both the app and paper group and during week 3 and 4, the adherence in the app group was significantly lower than the adherence in the paper group. Contradictory results were found by Wall et al., as they showed a trend towards higher adherence in the app-supported group compared to the home practice group [19]. In addition, also the study of Starmer and colleagues, working with an application as an adjunct to standard therapy, showed discrepancies with our results [22]. The lower adherence rates found in the app group compared to the paper group might be explained by the not so much appreciated game element in the app. Some patients indicated that it was too childish, others stated that they did not pay much attention to it. Another explanation might be the impractical way of performing exercises with the tablet, leading to little motivation; since the tablet was not directly linked to the exercise devices, patients had to enter the performed exercises themselves during practice.

In addition to the higher adherence rates found in the therapist group, there were clearly less dropouts in this group, supporting the idea of higher motivation.

Our study is however not without limitations. First of all, adherence data in both the paper and app group rely solely on participant information, which could possibly create a bias. During the study, we understood that some patients don’t want to disappoint the SLP by admitting they didn’t practice (enough). This may have led to a false record of adherence and thus a presumably slightly lower adherence than reported above. Second, only 13 of the 46 patients in the app group were questioned about their experiences with the app, making it difficult to build any firm conclusions based on this data.

Since our results suggest higher adherence rates to PSE in patients continuously supported by the therapist, it might be interesting to find out if these results can also be achieved when practicing more than 4 weeks. However, no clear guidelines exist in when the exercises should start and how long they should last in time. In this study, we practiced only the first 4 weeks since literature shows that feasibility of completing PSE decreases during the last treatment weeks and the aim of this RCT was to optimize adherence rates [2, 36]. Nonetheless, the question arises if practicing the 4 four weeks is enough to achieve benefits on swallowing function, muscle strength and QoL. Future research should focus on the effects of differences in timing and duration of PSE and of course, if therapist supervision could lead to high (or acceptable) adherence rates during the last weeks of RT/CRT.

In this study, the paper group achieves high adherence rates during the first two weeks of RT/CRT, without a significant difference compared to the therapist group, and moderate rates during week 3 to 4. Based on these results, a possible solution to improve adherence might be an increase in supervised sessions towards the end of exercise weeks, as our findings suggest that stimulation and motivation by an SLP is most needed in the last exercise weeks. A combination of home practice the first two weeks with face-to-face exercises afterwards is subject for further research. In addition, future research should also focus on whether practicing from home, but with daily therapist interaction via an app, would achieve the same results as practicing with an SLP in real life.

The next steps within the PRESTO study are to examine the effects of increased adherence on muscle strength, swallowing function and QoL, possible confounding factors and the cost-effectiveness.

In conclusion, our randomized controlled trial found significant differences in adherence rates to PSE based on service-delivery mode in HNC patients undergoing RT/CRT. Highest rates were found in the therapist group while adherence was moderate to low in the paper and app group, respectively. Based on these findings, we can conclude that increasing the face-to-face contact with a SLP can be the solution to the well-known problem of low adherence to PSE in this patient population.

## Data Availability

The datasets generated during the current study are not publicly available since they contain patient data and the Informed Consent does not include sharing data publicly. They are available from the corresponding author upon reasonable request. All clinical record forms are collected and managed using REDCap (Research Electronic Data Capture) electronic data capture tools hosted at Ghent University Hospital (1).
